# Prediction of prostate cancer biochemical recurrence by using discretization supports the critical contribution of the extra-cellular matrix genes

**DOI:** 10.1038/s41598-023-35821-1

**Published:** 2023-06-22

**Authors:** Laura Marin, Fanny Casado

**Affiliations:** 1 Department of Engineering, Pontificia Universidad Catolica del Peru, Av. Universitaria 1801, San Miguel, 15088 Lima, Peru; 2 Institute of Omics Sciences and Applied Biotechnology, Pontificia Universidad Catolica del Peru, Av. Universitaria 1801, San Miguel, 15088 Lima, Peru

**Keywords:** Computational biology and bioinformatics, Molecular medicine

## Abstract

Due to its complexity, much effort has been devoted to the development of biomarkers for prostate cancer that have acquired the utmost clinical relevance for diagnosis and grading. However, all of these advances are limited due to the relatively large percentage of biochemical recurrence (BCR) and the limited strategies for follow up. This work proposes a methodology that uses discretization to predict prostate cancer BCR while optimizing the necessary variables. We used discretization of RNA-seq data to increase the prediction of biochemical recurrence and retrieve a subset of ten genes functionally known to be related to the tissue structure. Equal width and equal frequency data discretization methods were compared to isolate the contribution of the genes and their interval of action, simultaneously. Adding a robust clinical biomarker such as prostate specific antigen (PSA) improved the prediction of BCR. Discretization allowed classifying the cancer patients with an accuracy of 82% on testing datasets, and 75% on a validation dataset when a five-bin discretization by equal width was used. After data pre-processing, feature selection and classification, our predictions had a precision of 71% (testing dataset: MSKCC and GSE54460) and 69% (Validation dataset: GSE70769) should the patients present BCR up to 24 months after their final treatment. These results emphasize the use of equal width discretization as a pre-processing step to improve classification for a limited number of genes in the signature. Functionally, many of these genes have a direct or expected role in tissue structure and extracellular matrix organization. The processing steps presented in this study are also applicable to other cancer types to increase the speed and accuracy of the models in diverse datasets.

## Introduction

Gleason score, a long-established approach to determine the aggressiveness of prostate cancer, relies exclusively on the architecture and morphological variance of tissue structures with the purpose of proposing suitable therapeutic strategies. However, using the same approach to monitor after therapy and during remission might not be as informative. Indeed, about 20% to 30% of men will relapse and experience biochemical recurrence (BCR) with varied courses of action available^1^. Latest advancements on RNA-sequencing have improved our understanding of cancer biology, and certain genes have been proposed to predict the chance of presenting biochemical recurrence. Zhang H et al.^2^ worked with the expression of one single gene, the RABEX- 5; and its implication in the biochemical recurrence. Meanwhile, Chu et al.^3^ used the expression of eight genes to detect patients with high risk of biochemical recurrence. Comparably, Zhao L^4^ constructs a model to predict BCR with the three following genes CA14, LRAT, and MGAT5B. Nonetheless, finding clinically relevant biomarkers has been proven to be highly complex for prostate cancer, mostly due to limited robustness^5^.

Discrete genetic expression has improved tremendously the performance of machine learning algorithms and can address the current limitations to isolate the genes responsible for recurrence, and to confine the expression of the genes into intervals for a better understanding of prostate cancer mechanisms. Data discretization, while not widely used in studies of prostate cancer and risk of biochemical recurrence, is a prominent tool in Statistics. In Ref.^6^, equal frequency binning discretization of RNA-seq data was employed to classify four known subtypes of glioblastoma multiform and improved significantly the classification by creating a more robust model with a reduced number of variables in the final signature. For the purpose of predicting the recurrence of contrasting types of cancer in a five-year time-lapse, Shoon^7^ reports a machine learning based approach operating on a pre-selected and entropy minimization discretized microarray. The suggested procedure, with an average accuracy of 98.9% in predicting recurrence, demonstrates the efficiency and cost-effectiveness of a discretized microarray.

By definition, discretization transforms a continuous-valued variable into a discrete one by creating a set of contiguous intervals cut-points spanning the range of the values of the variable of interest. Converting continuous biological data into discrete data with finite values, decreases the degrees of freedom of the data, while enhancing the overall interpretability of the results, creating a more accurate model^8^. Discrete values facilitate the comprehension of the variables while boosting the correlation between the attributes and the target variable^9^. Linear machine learning models benefit from discretization, by reducing the representation bias generated when dealing with non-linear datasets, and enabling the deduction of several decision boundaries within a single model^10^. Discretization optimizes the learning process and it enhances knowledge reduction because noise presented in extensive datasets is diminished^11^. Given the different discretization approaches to choose when developing a method, our work focuses on minimizing information loss during the transition to discrete values from previously continuous normalized gene expression values of prostate cancer samples. The main goal of this study is to evaluate the application of discretization in prostate cancer genomics by comparing two unsupervised discretization approaches, with the purpose to deduce the optimal number of intervals for each gene. This study suggests a model capable of determining with high accuracy a reduced subset of genes and the prostate-specific antigen (PSA) levels responsible for the early and advanced biochemical recurrence, by isolating the genes responsible for the recurrence in contrast to overall expression. The final gene signature may improve the quality of life of patients by predicting the risk of presenting biochemical recurrence within the two-years after their final treatment.

## Results

### Patient status classification with optimal number of intervals for discrete data with small number of genes

Across the log2 normalized TCGA genome, some expression levels did not vary between cases. Attributes with a coefficient of variation below 1.5 were removed. We prospected a distinct number of bins with the remaining 7800 normalized from [0,1] genes, according to the rules from Table 1. Then, a subset of genes was selected in the TCGA dataset according to their correlation with the target status. The logistic regression was then trained with the specific genes template, tested with GSE54460 and MSKCC and the final signature was validated using the GSE70769 dataset.

**Table 1:**
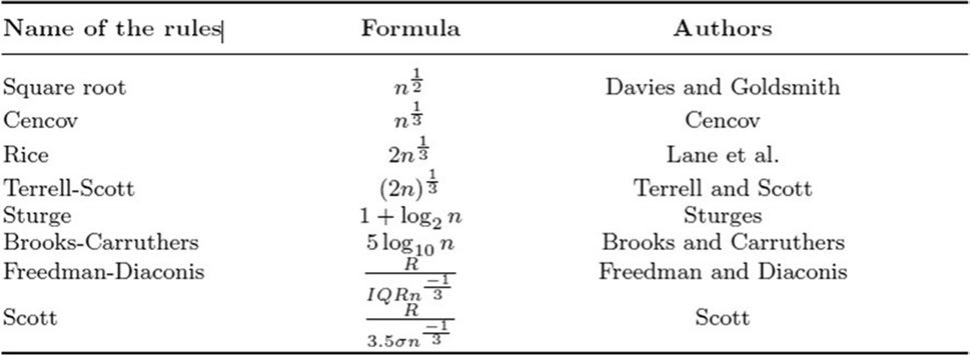
Table of methods to estimate the number of discretization.

Overall, equal width discretization proffered classification with higher accuracy with a limited number of genes as displayed in the Tables 2 and 3. It was difficult to choose the appropriate discretization as demonstrated above since an equal width discretization of eight and eleven, delivered inferior results than without discrete variables.

**Table 2:** Comparison of methods to estimate patient status with the smallest number of genes and higher accuracy with equal WIDTH discretization.

Rules	Number of bins	Accuracy	Precision	Number of genes
Without discretization	0	75%	75%	19
Square root	12	77%	79%	31
Cencov	5	82%	82%	10
Rice	10	77%	75%	7
Terrell-Scott	6	79%	79%	26
Sturge	8	71%	74%	21
Brooks- Carruthers	11	73%	79%	29
Freedman-Diaconis	Dynamic	73%	74%	37
Scott	Dynamic	72%	74%	42

**Table 3:** Comparison of methods to estimate patient status using the smallest number of genes and higher accuracy with equal FREQUENCY discretization.

Rules	Number of bins	Accuracy	Precision	Number of genes
Square root	12	75%	77%	31
Cencov	5	77%	77%	38
Rice	10	71%	71%	16
Terrell-Scott	6	78%	78%	39
Sturge	8	75%	76%	41
Brooks-Carruthers	11	77%	77%	37
Freedman-Diaconis	dynamic	71%	72%	77
Scott	dynamic	72%	73%	63

A five equal width discretization, achieved the best classification of the patient status with an 82% accuracy, and a ten-genes signature. Out of the 51 patients with BCR in the GSE54460 testing dataset, 47 were classified correctly, by the model according to the Eq. (1). Classification reached 75% when applied to an unfamiliar validation dataset (GSE70769).

From the gene signature in 1, ZFHX3, NFIB, PCCA, WDR5 are predominantly over-expressed in patients without BCR, while AIDA is under expressed. On the other hand, under expression of SLC25A30 and ITPR1 evidences risk of BCR. The probability for the patient to present BCR is displayed in the signature below. When the genes expression of the patients falls into the designated range, their associated weights are summed and final score pass through the following formula.

$$ {\text{Probability}}\,{\text{of}}\,{\text{BCR}}\, = \,{\text{e}}_{{{\text{final}}\,\,{\text{score}}}} /\,\left( {{\text{e}}_{{{\text{final}}\,\,{\text{score}}}} + {\text{ e}}_{{{\text{final}}\,{\text{score}}}} } \right) $$

1 $$ {\text{Class Progressed}}:{ 1}.{5}\, + \,\left[ {{\text{ZFHX3}} =^{\prime } (0.{8} - {1})^{\prime } } \right] \times - \,0.{73}\, + \,\left[ {{\text{EMP2}}\, = \,^{\prime } \left( {0.{6} - 0.{8}} \right]^{\prime } } \right]\, \times \, - \,0.{49}\,\, + \,\left[ {{\text{ITPR1}}\, = \,^{\prime } \left( {0.{2} - 0.{4}} \right]^{\prime } } \right]\, \times \,0.{66}\,\, + \,\left[ {{\text{NFIB}}\, = \,^{\prime } \left( {0.{6} - 0.{8}} \right]^{\prime } } \right]\,\, \times \, - \,0.{57}\,\, + \,\left[ {{\text{PCCA}}\, = \,^{\prime } \left( {0.{6} - 0.{8}} \right]^{\prime } } \right]\,\, \times \, - \,0.{73}\,\, + \,\left[ {{\text{RGS2}}\, = \,^{\prime } \left( {0.{4} - {1}} \right)^{\prime } } \right]\, \times \, - \,{1}.0{6}\,\, + \,\left[ {{\text{W DR5}}\, = \,^{\prime } \left( {0.{8} - {1}} \right)^{\prime } } \right]\, \times \, - {1}.{15}\,\, + \,\left[ {{\text{SRGAP2}}\, = \,^{\prime } \left( {0.{2} - 0.{4}} \right]^{\prime } } \right] \times \, - 0.{48}\,\, + \,\left[ {{\text{AIDA}}\, = \,^{\prime } \left( {0.{4} - {1}} \right)^{\prime } } \right]\, \times \,0.{57}\, + \,\left[ {{\text{SLC25A3}}0 \, =^{\prime } (0 - 0.{2}]^{\prime } } \right]\, \times \,0.{84}{\text{.}} $$

### Predicting time of recurrence with optimal number of intervals for discrete data using a small number of genes

**Figure 1:**
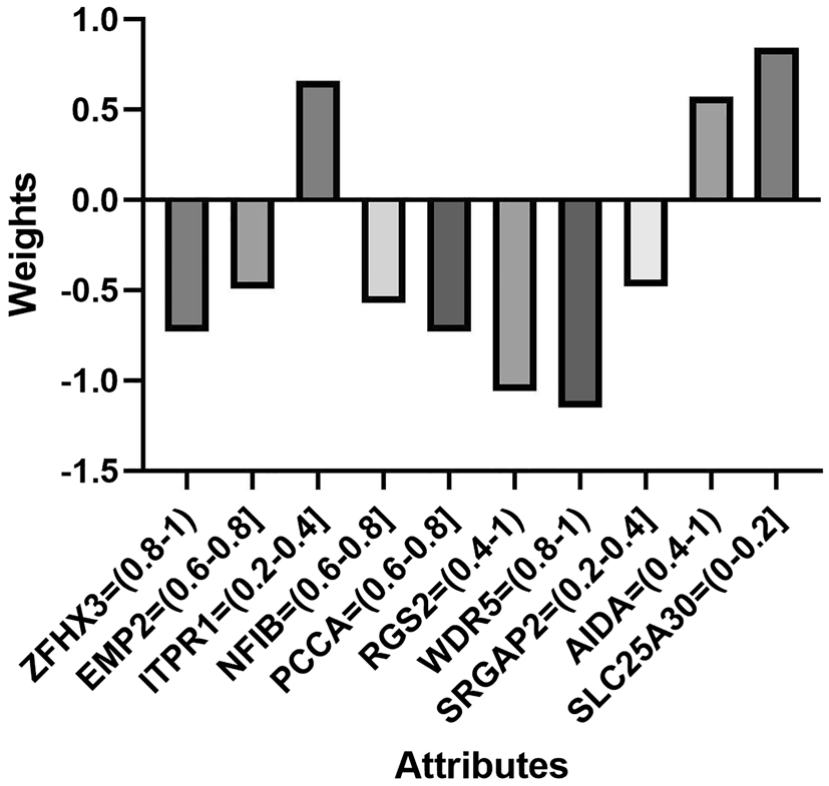
Genes and their respective intervals associated with weights involved in the logistic regression signature to predict the risk of BCR.

**Figure 2:**
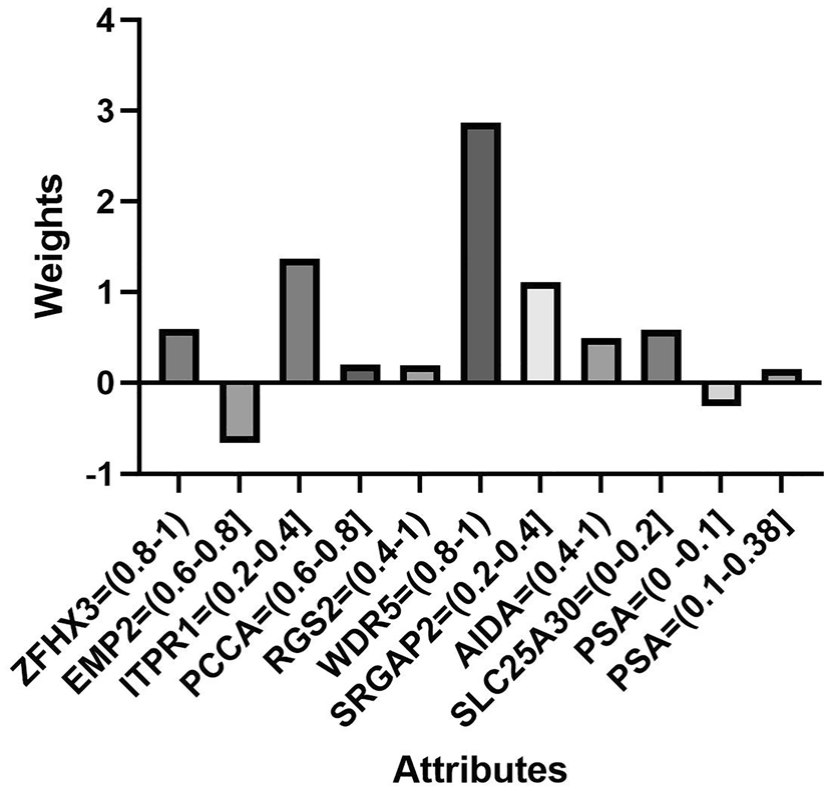
Genes and their respective intervals associated with weights involved in the logistic regression signature to predict the risk of BCR within a two-years time-lapse.

To predict the time of recurrence for patients with BCR through the 10 confirmed genes the TCGA patients were divided into two equals groups (35 samples with BCR two years after treatment, and 36 without) and the 10 discretized genes expression (Fig. 1). These data were passed through logistic regression and tested on the remaining 83 BCR patients. Nonetheless, prediction of BCR proved to be a much more complex task. In consequence, beside genetic expression, the PSA levels were also added to the equation shown in Fig. 2.

The ten-genes signature with PSA levels predicted early BCR with an accuracy of 71% in the testing dataset and 69% on the validation dataset as disclosed in Table 4. Furthermore, a PSA level below 0.38 ng/ml was predominantly associated with a risk of BCR within a two-years time-lapse. From both gene signatures, EMP2, ITPR1, AIDA and SLC25A30 can be identified as evident predictors of recurrence. Genes ITPR1 and SLC25A30 are under-expressed in both gene signatures, whereas AIDA is over expressed. Also, high expression of EMP2 exhibits low risk of recurrence, as displayed in Fig. 2.

**Table 4:** Results obtained when adding the PSA level to the 10 discretized genes expression.

Model evaluation	Testing group		Validation group	
	0–24 months	> 24 months	0–24 months	> 24 months
Precision	0.711	0.733	0.687	0.675
ROC area	0.68		0.64	

**Figure 3:**
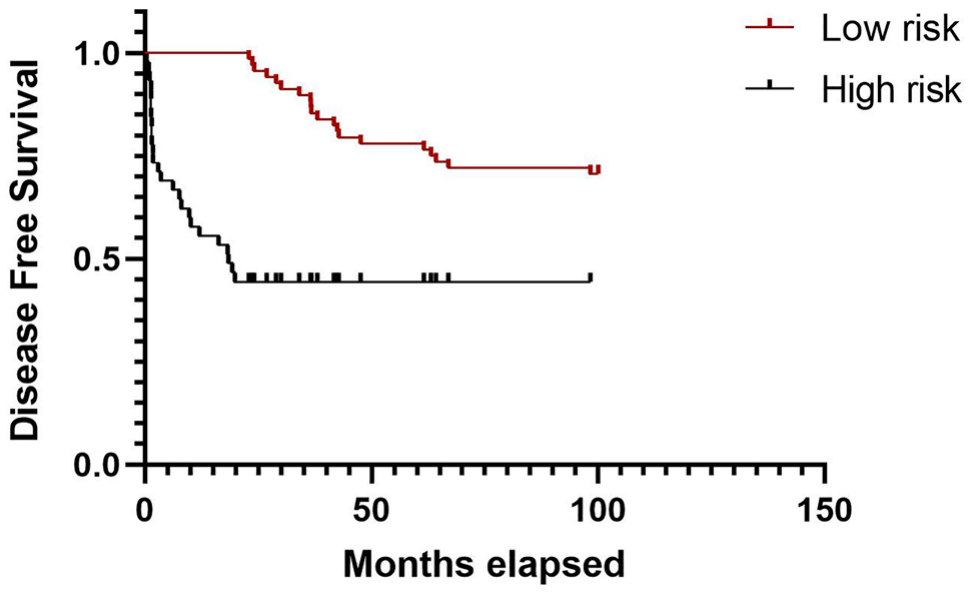
Kaplan–Meier curves to estimate the patients’ recurrence from the 10 genes signature for the validation test.

The probabilities for patients in the validation test to present BCR are displayed in the Kaplan–Meier plots 3 (Fig. 3), where high risk versus low risk groups are divided according to the risk score from the genes signature. High risk is defined for patients who did not present BCR during the study, or a disease free time over 24 months.

## Methods

### Training, testing and validation datasets

Prostate genetic expression is accessible to the public from the Program The Cancer Genome Atlas (TCGA) data portal. We used the Prostate Adenocarcinoma (TCGA, PanCancer Atlas) dataset published at https://www.cbioportal.org/study/summary?id=prad_tcga_pan_can_atlas_2018. Along with the genomics expression, clinical information including age, race and disease- free time as well as whole slide images from 500 patients are available in the platform. Out of the 500 patients, 81 patients present BCR information, however only 71 were retained since they included complete clinical information. From the 419 patients that do not present recurrence, 72 were selected to compensate the dataset since an unbalanced cohort would affect negatively the final model. To choose patients without BCR in the dataset, patients with similar time to recurrence were kept. We assumed that by working with patients with different diagnosis but similar time to BCR, a more accurate signature genes to predict the recurrence of the prostate cancer can be found. Altogether, the training dataset from TCGA includes 143 patients, 72 of them do not present recurrence, while 71 present a BCR. In addition, a testing dataset was established with data from the Memorial Sloan Kettering Cancer Center (MSKCC) https://www.ncbi.nlm.nih.gov/geo/query/acc.cgi?acc=GSE21032 containing information from 36 patients with recurrence, and 104 without BCR. Finally, data from the GSE54460 https://www.ncbi.nlm.nih.gov/geo/query/acc.cgi?acc=GSE54460 was incorporated to include 45 additional patients with recurrence. A validation data set was created incorporating 93 patients, their corresponding PSA level and time of recurrence.

The following Table 5 summarizes conformation of the datasets.

**Table 5:** Number of patients included in the training and testing datasets.

	Training group	Testing group	
	TCGA	MSKCC	GSE54460
With BCR	71	36	55
Without BCR	72	104	0
Median follow-up			
Time (months)	44.7	45.5	23.0
Age (years)			
≤ 65	101	117	0
> 65	42	23	0
Not available	0	0	45
Gleason score (%)			
≤ 7	72	127	36
> 7	71	13	9
PSA level (ng/mL)			
≤ 10	5	114	18
> 10	138	24	26
Not available	0	2	1
Tumor stage (%)			
T1/T2	47	86	25
T3/T4	126	54	20

The raw TCGA dataset comprising the expression of more than 20,000 genes was first normalized by applying the base 2 log. Once normalized, genes with a variation above 1.5 are retained, with a final set of 7800 genes. The same genes were selected in the testing datasets. To ensure the equity of each attribute, normalization is applied to the training and testing dataset, where the minimum E_m_in value gets converted to 0, the highest E_m_ax to 1 and the gene expression in the n row follows

$$ {\text{Normalized}}\,\left( {{\text{value}}_{n} } \right)\, = \,\frac{{{\text{e}}_{{\text{n}}} \, - \,{\text{E}}_{{{\text{min}}}} }}{{{\text{E}}_{{{\text{max}}}} - {\text{E}}_{{{\text{min}}}} }}. $$

### Discretization

Different studies to estimate the ideal number of bins have used the formula (1). Out of the eight rules, the first six are stable since they only depend on the number of attribute n. Concurrently, Freedman and Scott include the difference between the maximum and minimum values or the range of the dataset (R). The interquartile range (IQR) describes the middle 50% of values from the lowest to the highest one and σ represents the standard deviation of each attribute. The following table shows the discretization approaches considered in this study.

Equal frequency binning, divides the attributes into n number of intervals, containing the same number of values. Equal width subtracts the maximum value from the minimum value of each feature, divides it by the number of bins,

$$ {\text{width}}\, = \,\left( {{\text{maximum}}\,{\text{value}}\, - \,{\text{minimum}}\,{\text{value}}} \right){\text{/number}}\,{\text{of}}\,{\text{bins,}} $$

to create intervals of equal size.

### Feature selection

We selected a subset of relevant discrete attributes associated with logistic regression to reduce the risk of over-fitting, and therefore to improve computational efficiency by removing irrelevant features embodying noise in the models^12^. Feature selection algorithms established on the correlation between the patient status and their attributes was used to retrieve the most favorable gene template for the prediction.

The Correlation-based Feature Selection (CFS) algorithm rank the features according to their correlation with the target attribute. With this intent, the merit score of S subset including l feature is calculated by *Merit score* = $\frac{lt_c}{\sqrt{l+l(l-l)t_f}}$ where *t*_*f*_ the correlation score between 2 features, and *t*_*c*_ the correlation value between features and patient group. Genes with a merit score above 0.60 are retained to train the model, while the rest are removed from the dataset.

### Logistic regression

Logistic regression alone was considered in the methodology because it may confer fewer variables in the end signature, a desirable characteristic when minimizing diagnostic cost and traceability. While a decision tree might have increased the overall classification, the summation of the nodes may complicate the interpretability of the results^13^. However, logistic regression favored the comprehension of the influence of each variable to the final model of patient outcome. Additionally, higher accuracy can be reached by training neural networks, nonetheless, the different layers of neuron may prevent the proper analysis of the genes responsible for the recurrence^14^.

Our approach focused on binary logistic regression in order to classify the patient as recurrent or non-recurrent or with a time of recurrence above or below 24 months.

Logistic regression, like the linear regression represented by

$$ {\text{Z}}\, = \,{\text{wx}}\, + \,{\text{b,}} $$

was represented as an equation^15^. Each input values was combined using weights (w and b in the equation) to predict the output y. In the case of logistic regression, the model is represented by: ${\text{y}}\,{ = }\,{\text{e}}^{{\left( {{\text{b}}0\, + \,{\text{b1}}\, \times \,{\text{x}}} \right)}} /\left( {1\, + \,{\text{e}}^{{\left( {{\text{b}}0\, + \,{\text{b1}}\, \times \,{\text{x}}} \right)}} } \right)$ with y being the output value, x the input one and b0 and b1 the associated weights. The main goal is to determine the b weights, with sigmoid cost function, in order to predict correctly the output value. L2 regularization is tuned to shrink the weights towards zero, thus reducing the variance of the model and avoiding over-fitting in the training set, and the solver liblinear enforced. The logistic regression can be also used on a case of a classification by replacing the nominal value by numeric value.

### Model evaluation

The logistic models were evaluated with a ratio between the number of instances correctly classified, divided by the total number of instances, a concept also known as accuracy. Additionally, the precision, defined as how close the model prediction is to real observations, was calculated as explained here:

$$ {\text{Precision}}\, = \,{\text{True}}\,{\text{Positives}}\,/\,\left( {{\text{True}}\,{\text{Positives}}\, + \,{\text{False}}\,{\text{Positives}}} \right). $$

The Receiver Operating Characteristic (ROC) curve is a graphic summarizing the performance of the model by representing the true positives and the true negatives. The Area Under the Curve (AUC) was also calculated, where a higher score imply superior classification; nevertheless, Area under the score of 1, may also indicate over-fitting.

### Ethical compliance

This research did not involve studies involving animal or human participants. Public datasets were employed. No specific permissions were required for corresponding locations.

## Discussion

We propose a machine learning algorithm, data pre-processing and features selection to classify if and when prostate cancer patients will present BCR, while highlighting the range of gene expression accountable for the recurrence. In the final model, equal width binning outperformed equal frequency and normalized datasets in both accuracy and number of variables. The five- bins discretization provided the best model to classify the patients according to their status, and predict recurrence within the first two-years after last treatment.

Predicting the actual genes responsible for the recurrence of the cancer can be a heavy task due to the characteristic of the genes. However, by discretizing the data, and gathering patients with similar biochemical recurrence time, we might be able to provide better care and treatment to the patients, through ten-genes signature: ZFHX3, EMP2, ITPR1, NFIB, PCCA, RGS2, WDR5, SRGAP2, AIDA, SLC25A30 selected with an accuracy of 82%. Studies such as Ref.^16^ achieved a four-genes signature classification with 83% of accuracy, meanwhile the investigation led by Ref.^17^, isolated twelve genes overexpressed in patients with BCR. Equivalently, the multivariate cox Regression from Ref.^18^, showed a five-genes signature and displays an Area Under the Curve of 0.62 for validation cohort, correlating positively the genes ORM1, DDC and LINC01436 to BCR, while the remaining two genes from the signature, AOC1 and PAH were negatively associated with BCR. Even though different sets of genes were found in these different studies, altogether they share functional roles related to extracellular matrix (ECM) and cell proliferation. This is consistent with ECM´s role to provide a structured environment for the tissue, warranting the organization of the glands, modulating cell migration and proliferation. Changes in its composition have been associated with tumor cell migration and presence of metastasis^19,20^.

This work contributes with new evidence of a link between changes in expression of certain genes and recurrence of prostate cancer using discretization approaches. The biological plausibility of our results is supported by previous data showing diverse relationships between prostate cancer and some of these genes. Rui^21^ developed a ten-genes signature based on tumor- adjacent normal tissue. The isolated genes were directly linked to cell-to-cell signaling, a component of the extracellular matrix^22^. Furthermore, the level of WDR5 expression is higher in prostate cancer than healthy prostate tissue; its interaction with androgen signaling, infers its purpose in the acceleration of prostate cancer cell proliferation^23^. Patients with greater levels of ZFHX3 correlate with better survival^24^, as loss of ZFHX3 increased cell proliferation^25^. Previous experimentation performed in mice demonstrated that ZFHX3 acted as a tumor suppressor in prostate cancer, as its reduced expression disrupts the proper organization of the glands, affecting the layer of muscle between the stromal and epithelial cells^25^. ITPR1 although not frequently mentioned in prostate cancer, directly affects the process of apoptosis in colorectal cancer and ovarian cancer^26^. Its depletion can increase the loss of apoptotic control, hence prolonging the survival time of cancer cells. Reduced NFIB expression causes prostate hyperplasia, due to prostate gland enlargement^27^, while not directly associated with prostate cancer, they can be understood as a consequence of a disturbed ECM.

Cohorts from available datasets commonly comprise more patients without BCR. An unbalanced dataset can, as a matter of fact, create a bias towards majority class samples^28^. Most prostate cancer relapsing prediction either overlooks this issue^4,29^ or employs balancing techniques such as oversampling, under-sampling or hybrid^30–32^. Oversampling implies copying minority classes, discarding variant expression, while under-sampling might delete useful information. Integration of multiple types of data, not only adds more variables to the study, but also aims for higher accuracy, robustness and greater statistical power, while assessing results from the model. Thus, our approach takes into consideration molecular changes, and PSA levels across patients.

The implementation of discretization allows the prediction of the actual time of recurrence within two-years time period while isolating a template of ten-genes. The quantitative expression of the genes can therefore be analyzed, and acknowledged as biomarkers. Within the experiment, the number of genes involved in the signature varied and their accuracy was related to the chosen number of bin and discretization strategies. Due to their complexity, correlation between the gene expression and their target might be indiscernible for the model. Lower bin numbers simplify the dataset and its interrelationship, but they may overlook functional information, enlisting alternatives of genes to compensate the loss. In contrast, an elevated bin number prevents the recollection of said interdependence. In summary, the power of the model to predict varies according to the discretization approach applied.

While our results focus on patients classified by our methodology with higher risk of BCR within the two years after being declared free of cancer^33,34^, classified the patients between low to high risk, with a variation in between the class that can vary from three to five years. Increased prediction precision will benefit the patients, and increase their overall life expectancy, hence maintaining them under active surveillance. Biomarkers can then be established not solely for the risk of showing recurrence, but additionally for the early signs of BCR. The gene signature to predict the time of recurrence relies exclusively on the same ten genes, suggesting their extensive importance in the recurrence and manifest the role of the ECM, while PSA was evidenced as a powerful variable in BCR prediction. The importance of the ECM in BCR, was only recently settled^35^, but causal research remains at its early stages. Additional work needs to focus on the phenotype of the biomarkers expressed by the outlined genes in order to narrow down the interval of prediction, and strengthen the ECM components responsible for BCR, along with proposing indicators of the aggressiveness of the cancer.

## Data Availability

The datasets analysed during the current study are available in the The Cancer Genome Atlas (TCGA) data portal: the Prostate Adenocarcinoma, https://www.cbioportal.org/study/summary?id=prad_tcga_pan_can_atlas_2018; the Prostate Cancer dataset from the Memorial Sloan Kettering Cancer Center (MSKCC), https://www.cbioportal.org/study/summary?id=prad mskcc; and datasets published at the National Center for Biotechnology Information (NCBI) portal with accession number GSE54460, and GSE70769 https://www.ncbi.nlm.nih.gov/geo/query/acc.cgi?acc=GSE54460; https://www.ncbi.nlm.nih.gov/geo/query/acc.cgi?acc=GSE70769.
